# Ichthyosis follicularis, alopecia, and photophobia (IFAP) syndrome

**DOI:** 10.1186/1750-1172-6-29

**Published:** 2011-05-21

**Authors:** Hala Mégarbané, André Mégarbané

**Affiliations:** 1 Service de Dermatologie, Saint Georges Hospital, Beirut, Lebanon; 2 Unité de Génétique Médicale et Laboratoire Associé INSERM UMR_S910, Université Saint-Joseph, Beirut, Lebanon

**Keywords:** IFAP, Genodermatosis, X-linked, *MBTPS2 *gene

## Abstract

The IFAP syndrome is a rare X-linked genetic disorder reported in nearly 40 patients. It is characterized by the triad of Ichthyosis Follicularis, Alopecia, and Photophobia from birth. Other features such as short stature, intellectual disability, and seizures may develop in the first few years of life. Skin histopathology is non-specific and consists of dilated hair follicles with keratin plugs extending above the surface of the skin, decreased or absent sebaceous glands, and decreased desmosomes in number and size. The disorder results from mutations in the *MBTPS2 *gene that impairs cholesterol homeostasis and the ability to cope with endoplasmic reticulum stress. Follicular hyperkeratosis can be treated using topical keratolytics, emollients and urea preparations. A moderate response to acitretin therapy has been noted in some patients. Intensive lubrication of the ocular surface is essential. Life expectancy in patients with IFAP syndrome can vary from death in the neonatal period to normal surviving. Cardiopulmonary complications remain the major cause of death.

## Disease name and synonyms

Ichthyosis Follicularis, Atrichia, Photophobia

IFAP syndrome

## Definition

IFAP syndrome (OMIM 308205) is a rare genetic disorder characterized by ichthyosis and alopecia from birth and sometimes accompanied by short stature, intellectual disability, and seizures that develop in the first few years of life. Photophobia may also be present in the first year of life or appears in infancy or early childhood. Its mode of inheritance is X-linked recessive, thus mostly affecting males. Affected or carrier females may display some of its clinical features.

## Epidemiology

The association of ichthyosis follicularis, atrichia, and photophobia was first reported as a syndrome by MacLeod in 1909 in three boys [1]. Since then a little more than 40 patients have been reported with also additional features (Table 1) [2-22].

**Table 1:** Review of clinical features associated with IFAP syndrome.

Clinical Feature	Percentage of male patients
Congenital alopecia	100

Developmental delay	32

Hypotonia	8

Short stature	25

Microcephaly	17

Frontal bossing	15

Photophobia	100

Dystrophic nails	40

Seizures	28

Intellectual disability	39

Ichthyosis	100

Psoriasiform plaques	32

Cheilitis	24

Lack of sebaceous glands	46

Hypohidrosis	11

Hyperkeratosis	33

Spiny follicular projections	29

Atopic manifestations	36

Recurrent infections	32

Inguinal hernia	18

Vertebral malformations	25

Cleft hand	10

## Clinical description

**Figure 1:**
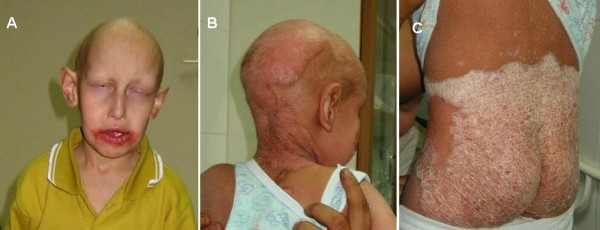
**Photographs of patients with typical features of IFAP syndrome**. Note: A) the atrichia, the photophobia, the cheilitis around the mouth, B) the ichthyotic scaling and erythematous and yellowish thick scaly hyperkeratotic plaques over the scalp, and C) the psoriasiform plaques over the buttocks.

All affected males have the IFAP triad of follicular ichthyosis, atrichia of the scalp, and photophobia (Figure 1A) (Table 1).

### Cutaneous manifestations

Ichthyosis follicularis is characterized by widespread non inflammatory thorn-like follicular projections. Dyskeratotic papules are most pronounced over the extensor extremities and scalp and are symmetrically distributed [23]. Congenital alopecia involving the scalp, eyebrows and eyelashes is another essential cutaneous manifestation of IFAP (Figure 1A). A noncicatricial complete body alopecia is also a classical feature. Variable degrees of a collodion membrane may be present in the neonate. Psoriasiform plaques (Figure 1B-C), angular cheilitis, periungueal inflammation, dystrophic nails, hypohidrosis, and atopic eczema can be present. The palms and soles are generally unaffected; one patient had a plantar keratoderma [3]. Affected or carrier females could present milder signs and symptoms such as cutaneous hyperkeratotic lesions that follow the lines of Blaschko, asymmetric distribution of body hair, and/or patchy alopecia, phenotype attributed to lyonisation [11].

### Ocular manifestations

Photophobia is an essential feature for the diagnosis of IFAP (Figure 1A). It can be present early in life or later in childhood. Superficial corneal ulceration and vascularization may lead to progressive corneal scarring and photophobia [24]. Males with IFAP have an inexorable progression of corneal vascularization and loss of vision [14]. Atopic keratoconjunctival inflammation, chronic tearing, cataract, horizontal nystagmus, astigmatism and myopia have been reported as well [24]. Slit lamp examination can show the presence of a diffuse punctate epithelial keratopathy with diffuse vascularizing keratitis and rare areas of partial corneal opacification next to areas with maintained corneal transparency [14]. The anterior chamber, lens and ocular fundus are usually normal.

Affected or carrier females could also present photophobia in the first year of life, and retinal vascular tortuosity [24].

### Neurological features

The most frequent neurological features in IFAP are intellectual disability, and seizures (Table 1). Other findings include olivo-cerebellar atrophy, malformation of the temporal lobes, mild inner cerebral atrophy, and hypoplasia of the corpus callosum [2,10].

### Miscellaneous

Other clinical features associated with IFAP syndrome consist of short stature, dysmorphic features such as frontal bossing, choanal atresia, and large ears. Intestinal anomalies such as omphalocele, Hirschsprung disease, congenital aganglionic megacolon, stenosis of the small intestine, and inguinal hernia, renal, cardiac and vertebral anomalies, and cleft hands have been reported [14,17]. Recurrent infections are often noted in IFAP syndrome. External genitalia are almost always normal; few cases presented with cryptorchidism [3,14,16,17], and one with a hypospadias [17]. Dental development is normal.

## Etiology

IFAP syndrome results from missense mutations in the membrane-bound transcription factor protease site 2 (*MBTPS2*) gene [17]. MBTPS2 is a membrane-embedded zinc metalloprotease that activates signaling proteins involved in sterol control of transcription and endoplasmic reticulum (ER) stress response [25,26]. It impairs cholesterol homeostasis and the ability to cope with endoplasmic reticulum stress. Functional studies on different mutations showed that patients with mutations that result in the lowest residual MBTPS2 activity had the most severe phenotypes [17]. Nevertheless, no clear phenotype/genotype correlation could be evidenced. Indeed, recently a Japanese patient with IFAP syndrome carrying the c.1286G > A (p.Arg429His) mutation in MBTPS2, was not as severely affected as the patients from a German family carrying the same mutation [16,17]. Furthermore, it was shown that the p.Asn508Ser mutation causes IFAP syndrome and a close allelic syndrome named "Keratosis follicularis spinulosa decalvans" [17,27]. Those observations raise the possibility that modifying factors might modulate the phenotype in this syndrome.

## Diagnosis

The diagnosis of the IFAP syndrome is based on the clinical features and on the presence of a mutation in the *MBTPS2 *gene.

## Histopathology

Skin histopathology is non-specific and consists of dilated hair follicles with keratin plugs extending above the surface of the skin, decreased or absent sebaceous glands and normal sweat glands. Transverse section of scalp biopsy can reveal abortive sebaceous glands in hair follicles [9]. The number of total hair follicles is not significantly decreased suggesting that the pilosebaceous hypoplasia might arise from impaired maturation during hair follicle morphogenesis [9].

On electron microscopy moderate spongiotic changes associated with partial disruption of the intercellular bridges, decreased desmosomes in number and size, and some dyshesion of the cells could be seen [21]. Examination of the cornea with EM can show reduced number of desmosomes in the corneal epithelium, dispersed bundles of tonofilaments and dilated intercellular gaps with segregated desmosome remnants [5]

## Antenatal diagnosis

IFAP syndrome cannot be detected prenatally by ultrasonography. If the mutation has been characterized in a carrier mother, prenatal diagnosis can be proposed. No cases of mosaicism have reported so far.

## Genetic counseling

A recessive X-linked pattern of inheritance has been established for IFAP. Therefore, the risk for a female carrier to have an affected son is 50%. The mutation might also arise in the patient *de novo*.

Recently, a mother and daughter [19], and 2 unrelated female patients [4] with an IFAP syndrome were reported. They did not have linear distribution of skin lesions, suggesting an autosomal dominant mode of transmission. Thus, besides X-linked recessive inheritance, an autosomal dominant mode of inheritance could be present.

## Differential diagnosis

Generalized ichthyosis and alopecia have been reported in very few syndromes (Table 2). Among those, can be considered 4 diagnoses: the dermotrichic syndrome [28], hereditary mucoepithelial dysplasia (HMD) (OMIM 158310), Keratitis-Ichthyosis-Deafness syndrome (KID) (OMIM 242150), and keratosis follicularis spinulosa decalvans (KFSD) (OMIM 308800), the other ones being at variance with the IFAP syndrome.

**Table 2:** Major conditions in which ichtyosis and alopecia are both present (14).

SYNDROME	INHERITANCE	MIM
Alopecia-Skeletal anomalies-Mental retardation	Autosomal recessive	203550

Dermotrichic	X-linked recessive	308205

Ectodermal dysplasia-Alopecia-Mental retardation	Autosomal recessive	203550

Hay-Wells syndrome	Autosomal dominant	106260

Hayden syndrome	Uncertain	Reference 24

Hereditary mucoepithelial dysplasia	Autosomal dominant	158310

IFAP	X-linked	308205

Ichthyotis-Hypotrichosis-Hypohidrosis	Autosomal recessive	602400

Keratitis-Ichthyosis-Deafness (KID)	Autosomal dominant	242150

Keratosis follicularis spinulosa decalvans	X-linked	308800

Ichthyosis, alopecia, eclabion, ectropion and mental retardation	Autosomal recessive	242510

Trichooculodermovertebral syndrome	Uncertain	601701

Woodhouse-Sakati syndrome	Autosomal recessive	241080

The IFAP syndrome and the dermotrichic syndrome have overlapping manifestations. Both are characterized by ichthyotic lesions and atrichia from birth, and short stature, intellectual disability, and seizures. They can be differentiated mainly on the basis of nail, skeletal, and intestinal anomalies, hypohidrosis, and megacolon present in the dermotrichic syndrome and ocular and respiratory disorders in the IFAP syndrome. In fact, overlap between both syndromes had already been noted in few patients [13,14] showing that both syndromes could be identical.

The HMD is an autosomal dominant condition which can be differentiated from IFAP by the presence of well demarcated erythema of the oral mucosa and a psoriasiform perineal rash, chronic erythematous macules and papules on palate and gingival and recurrent respiratory infections in infancy, cataracts in childhood, and fibrocystic lung disease in adulthood [27].

KID syndrome shares many features with IFAP. Nevertheless, in patients with KID syndrome nails are often dystrophic, teeth may be small or malformed, and ocular changes are usually observed during the 2^nd ^or 3^rd ^decade. In addition, there is a congenital hearing loss, palmoplantar hyperkeratosis with leather grain-like keratoderma is present but no follicular hyperkeratosis, and the mode of inheritance is autosomal dominant [23].

KFSD is X-linked recessive and causes follicular hyperkeratosis, hyperkeratosis of the calcaneal regions of the soles, scarring alopecia, absent eyebrows and eyelashes, and a corneal dystrophy with marked photophobia. Carriers may have mild manifestations. KFSD differs from IFAP syndrome in that the alopecia is not congenital and is progressively scarring, and that affected patients have milder phenotype than those with IFAP. Recently, mutations in the *MBTPS2 *gene were found in KFSD patients indicating that both IFAP and KFSD are within the spectrum of one genetic disorder with overlapping phenotypes [29].

## Management

A moderate response to acitretin therapy at a dose of 0.3 to 1 mg/Kg/day with improvement in cutaneous features and corneal erosions but no changes regarding alopecia and photophobia have been noted in some patients [11,15]. Otherwise, follicular hyperkeratosis can be treated using topical keratolytics, urea preparations, and emollients. Topical retinoids are not suitable because of their irritation. Corneal vascularization is relentless in affected boys and does not respond to topical corticosteroid therapy. Intensive lubrication of the ocular surface remains the mainstay of therapy [24]. Seizures must be treated accordingly.

## Prognosis

Life expectancy in patients with IFAP syndrome can vary from death in the neonatal period to normal surviving. The oldest reported patient was 33 years old [10]. Cardiopulmonary complications were the main cause of death.

## Consent

Written informed consent was obtained from the patient's parents for publication of this review and any accompanying images. A copy of the written consent is available for review by the Editor-in-Chief of this journal.

## Competing interests

The authors declare that they have no competing interests.

## Authors' contributions

The authors contributed equally to this review. They read and approved the final version of the manuscript.
